# Spiritual Experiences of Patients in the Cancer Trajectory: A Content Analysis

**DOI:** 10.4314/ejhs.v32i6.12

**Published:** 2022-11

**Authors:** Mahboobeh Khosravani, Nazi Nejat

**Affiliations:** 1 Department of Nursing, School of Nursing, Arak University of Medical Sciences, Arak, Iran; 2 Department of Nursing, School of Nursing, Arak University of Medical Sciences, Arak, Iran

## Abstract

**Background:**

Introduction: Patients with cancer as a life-threatening illness have needs in various dimensions. One of the most crucial dimension is spiritually. The present study was conducted to elucidate the spiritual needs of patients with cancer.

**Methods:**

A qualitative approach involving face to face semi-structured interviews conducted in Arak city, Iran. In total, 24 participants were recruited through purposive sampling. All interviews were recorded and transcribed verbatim. The data were analyzed using content analysis.

**Results:**

Patients' experiences of spirituality were classified into four themes, including spiritual needs (need to be in nature, need to communicate with others, need to perform religious practices, need for solitude and reflections, need to trust and security), strategies to address spiritual needs (physical activity, achieving inner peace, communication with the nurse or physician, doing dhikr (Zikr) and pray, reading the religious book and texts, making vows, paying attention to and loving others), reasons for not using spiritual resources (lack of belief in the healing powers of praying, believing in a better life after death), and impacts of spiritual beliefs (hope of Life, belief in divine destiny, trusting in God's support, accepting the divine test).

**Conclusion:**

Exploring the experiences, perceptions, and spiritual needs of cancer patients is of great importance in providing spiritual care as one of the critical aspects of holistic care. Nurses should be educated for assessing spiritual needs of patients to provide spiritual care.

## Introduction

Spirituality has been regarded as the essence of human existence. The World Health Organization (WHO) defines health in four dimensions: “Health is a dynamic state of complete physical, mental, spiritual and social well-being and not merely the absence of disease or infirmity” (1). Physicians must try to understand the patients' agony in the context of beliefs and cultural values (2).

They also want health-care professionals consider their spiritual concerns and to be treated and cared as a human with all dimensions of their health (3). Studies have demonstrated that spirituality and spiritual beliefs increase the patient's ability to cope with a disease, increase their recovery speed, and provide them more ability to overcome the disease (4,5).

Spiritual tendencies lead to understanding, cherishing, giving meaning, hope, serenity, compassion, diminishing loneliness, enhancing self-esteem, communication skills, psychological competence, and holism (6). While, disregarding spiritual needs leads to the individual engaging in internal conflicts, feelings of emptiness, despair in the face of deprivation, and psychological pressures (7).

Patients with cancer in particular are at a greater risk of spiritual distress when confronted with illness or end-of-life difficulties (8). Such spiritual crisis can be an opportunity for nurses to provide spiritual care (9) for them according to the patient's specific socio-cultural and physiological characteristics. Therefore, it seems that acknowledging the spiritual needs of patients and subsequently providing spiritual care in line with it is deemed as a treatment strategy reducing physical and psychological issues and increasing the client's satisfaction level (6).

Spirituality is rooted in individuals cultural and the historical background (9). Defining and identifying the patient's spiritual needs is a vital element in providing respectful cultural care (10). Most of Iranians are Muslims and adhere to religious principles and values as an integral part of life and death). Moreover, due to the specific and individual nature of the cancer experience, “spiritual needs are different for everyone and can change over time” (11). Findings of studies showed that educational level, pain intensity, reduced physical capacity of patients, life satisfaction, depression, power in family flexibility, quality of life, spiritual well- being, disease stage, time since diagnosis, hospitalization frequency religiosity, being an inpatient, and perceiving the cancer as incurable, satisfaction with care are variables correlated with spiritual needs of patients with cancer (3,12–20). Therefore, effective and regular spiritual assessment enables health care professionals to find out any unresolved concerns or needs in terms of spirituality and patients' religious coping limitations and identify those who are most at risk for spiritual distress (21). It has always been assumed that qualitative methods provide a deeper understanding of social phenomena than quantitative methods (22). Therefore, the present study was conducted to explore the spiritual needs of patients with cancer in Arak, Iran.

## Materials and Methods

A qualitative approach using content analysis was employed to facilitate a rich exploration of participant spiritual needs. After obtaining approval from the Ethics Committee of Arak Medical Sciences University, the potential participants were provided with study-related information and the confidentiality of their information. All the participants were joined voluntarily and they completed the informed consent. Participants also were made aware that findings to be made available to them at the end of study. Participants were assured that their information would be kept confidential and that the findings of the study would be reported in groups and anonymously.

Semi-structured and in-depth face to face interviews were conducted with 24 participants who were recruited from the tertiary cancer hospital in Arak city, Iran. The interviews were held in hospital by one of researchers in the present study. The interviews ranged from 45 to 90 minutes. All interviews were recorded digitally and transcribed verbatim. The transcripts were checked to ensure participants' expressions and responses were accurately recorded by another researcher. Participants were asked to answer the following questions: “What are your needs at the moment?”, “What are your concerns?”, and “What relieves your tension?”. Participants were asked to describe the meaning of the spirituality and talk about their spiritual needs. Exploratory questions, such as “Could you explain more?” or “What do you mean?” were also asked in the interview. The interviews continued based on the needs of the study until data saturation.

To analyze the data, MaxQDA 2018 was used for the data encoding and classification process. The deductive directed approach to content analysis based on Hsieh and Shannon (2005) was used (23). The steps including preparation the data, reading the transcripts, highlighting all text that refers to different area of spirituality, coding all highlighted text related to the topic, coding all highlighted text that could not be coded with initial coding scheme with new codes, and developing sub themes and themes (23) were done by the researchers.

**Ethical considerations**: The present research is the result of a research project approved by the Research Ethics committee of Arak University of Medical Sciences with the following ethics code: IR.Arakmu.Rec.1399.33. After acquiring the legal permits and acquainting the participants with the goals and significance of the research, their conscious written consent was attained. Participants were assured that their information would be kept confidential and that the findings of the study would be reported in groups and anonymously.

## Results

The majority of the participants (n=15) were female, aged from 23 to 70 years and undergoing chemotherapy (Table 1). Data analysis led to the formation of four themes relating to the spiritual needs, strategies to address spiritual needs, reasons for not using spiritual resources, and the impacts of spiritual beliefs; with 18 sub-themes identified in each (Table 2).

**Table 1:** Descriptive characteristics of the participants

Sex	No.(%)
Female	15(62.5)
Male	9(37.5)
**Age (years)**	
Mean±SD	43.5±11.8
Range (years)	23–69
**Marriage status**	
Married	21(87.5)
Single	3(12.5)
**Education**	
Illiterate	1(4.2)
Primary	7(29.2)
Diploma	12(50)
Academic	4(16.6)
**Occupation**	
Employed	11(458)
Retired	1(4.2)
Housewife	12((50)
**Type of cancer**	
Gastrointestinal	9(37.5)
Breast	5(20.9)
Lymphoma	2(8.3)
Lung	3(12.5)
Leukemia	3(12.5)
Others	2(8.3)
**Type of treatment**	
Chemotherapy	17(70.8)
Radiotherapy	6(25)
Surgery	1(4.2)

**Table 2:** Spiritual needs, themes and sub-themes

Themes	Sub themes
**Spiritual Needs**	Need to Be in Nature
	Need to Communicate with Others
	Need to Perform Religious Practices
	Need for Solitude and Reflection
	Need to Trust and Security

**Strategies to address Spiritual-Religious Needs**	Physical Activity
	Achieving inner peace
	Communication with the nurse or physician
	Doing Dhikr (Zikr) and Pray
	Reading the Religious book and Texts
	Making Vows
	Paying attention to and loving others

**Reasons for Not Using Spiritual Resource**	Lack of belief in the healing powers of praying
	Believing in a better life after death
**Impacts of Spiritual Beliefs**	Hope of life
	Belief in divine destiny
	Trusting in God's support
	Accepting the Divine Test

**Spiritual Needs**

The main theme consisted of four sub themes. The need to be in nature, the need to communicate with others, the need to perform religious practices, and the need for solitude, reflection, and need to trust and security.

**Need to be in nature**: Participants remarked leisure and nature trips as incredibly considerable factors influencing the human spirit in the battle against cancer: “*...Mountaineering gives me peace of mind*” (p3). “...*I wish our house was in the country or suburbs with lots of green spaces where I could feel uplifted and distracted*” (p1).

**Need to communicate with others**: Interacting and communicating with others was described as a source of comfort by many participants: “*...Communication with my companion, family, friends, and children make me happy*” (p9). “*...Talking to my family members gives me peace*” (p5). “...*I like to talk to my best friends, it gives me peace*” (p2).

**Need to perform religious practices**: Participants explained that religious practices are incredibly important to cope with their illness, psychological distress and feel relaxed: “...*I love attending religious ceremonies*” (p4). “...*Ever since I was diagnosed with cancer, I've been attending morning prayers every day*” (p5).

**Need for solitude and reflection**: The statements of the participants demonstrate their desire for silence and peace. They need sometimes to be isolated and alone with themselves to think about their true wishes, goals, feelings and interests: *“...Solitude and silence calm me down*” (p7), “... *when I'm alone, I comfort myself* ” (p8).

**Need to trust and security**: Participants stated that after being diagnosed with cancer, their need for having trust in God increased because they believed that God is the ultimate creator of human existence: “...*When I realized I had cancer, I thought on the first night of the grave, I clung to God with both hands*” (p6), “...*Facing cancer underscores the need to trust in God in patients*” (p9).

**Strategies To Address Spiritual Needs**

Physical activity, achieving inner peace, communicating with the nurse and physician, doing dhikr(Zikr) and pray, reading religious books or texts, making vows, and paying attention to and loving others are categorized as solutions to meet spiritual-religious needs.

**Physical activity**: Participants stated that exercising had a significant impact on reducing complications and accelerated their recovery process: “... *Physical activity, yoga and swimming have reduced the symptoms and complications of my disease.*” (p8).

**Achieving inner peace**: Participants believed that a person who faced with daily challenges and stresses seeks ways to relax and achieve inner peace: “...*I forgive others, so that God will forgive me*” (p6), “...*My earlier life was nice. I did not appreciate it enough and God did this to me, now I should thank god a lot and I know that God will heal me* ” (p10).

**Communication with the nurse or physician**: Most participants, described that communication with the medical staff and their bedside manner in dealing with patients helps them to cope with the cancer and its symptoms and complications. “...*Talking to medical staff, such as a nurse or doctor, reduce my pain greatly*” (p10), “...*For a patient, the doctor's manner is remarkably impactful, even more than medication*” (p12). “...*The greatest and best treatment for a cancer patient is nurse or physician empathy with patients* ” (p4).

**Doing dhikr (zikr) and pray:** Participants stated that praying plays a significant role in their recovery and healing, especially prayers of others: “...*I believe the pray of others are more helpful and I pray for others too*” (p13), “...*I say Zikr and call God to cure me through praying*” (p17), “... *I pray for others and ask others to pray for me*” (p15).

**Reading the religious book and texts**: Participants explained that they read the Holy Qur'an and text because they considered the Qur'an as a spiritual miracle to find peace: “...*Reading the Qur'an inspires me, and I always have a prayer book with me and read it*” (p18), “... *Whenever I am worried about my illness, I write the names of Allah(God) and the verses of Quran that I recite on a small piece of paper.* ” (p2).

**Making vows**: In Iranian society, people make various vows to fulfill their wishes. Some participants also stated that they vow to solve their problems and regain their health. “... *I decided to spend money for Imam Hussein's shrine if I recover from cancer* ” (p19).

**Paying attention to and loving others**: The participants stated that if someone wants to be forgiven by God for sins, must forgive others: *”...I forgive those who hurt me, .... then hope Khoda(Persian word for God) forgive me*. (p21).

**Reasons For Not Using Spiritual Resources**

The findings of this study indicated that not believing in praying, and believing in a better life after death are reasons for not using spiritual and religious resources.

**Lack of belief in the healing powers of praying**: Some participants stated that they do not pray and do not ask others to pray for them: *”...I don't need others' praying and I believe that praying will not help me” (p. 16), “...I feel that praying have no effect on my situation. It is a misconception that the power of praying can save me without any medical treatment”* (p17).

**Believing in a better life after death**: Some participants stated that they had no spiritual needs, never thought about their problems, and were unafraid of death since they considered it a step towards better life: “...*I have no spiritual needs and I do not worry about my future. If I die, I'm sure I'll be more comfortable in a nicer place*” p22), “...*I have no impressions or fears of death. I believe in life after death*” (p24), “... *I'd rather not turn to Khoda and beg Him not to let me die, ... I welcome death because I am going to in a better place”* (p26).

**Impacts of Spiritual Beliefs**

The study findings demonstrated that hope of life, belief in divine destiny (the divine will and decree), trusting in God's support and accepting divine test can be considered the impacts of spiritual beliefs.

**Hope of life**: From the participants' point of view, illness enhances hope of life and efforts to achieve aspirations: *”...ever since I became ill, my hope of life has increased” (p2), “...no matter how difficult is the circumstances, I don't lose my hope, and I remind myself that for at the end of the dark night, there is light” (p. 22)*

**Belief in divine destiny**: Some participants stated that God determines the destiny of each person and we should accept it. “... Whatever God wills, will be. God wanted me to get cancer” (p. 8), “I believe in divine destiny...so I try to give meaning to my illness” (p. 21).

**Trusting in God's support**: Some participants stated that God is a powerful source of support for them “...*I always tell God that I have entrusted myself to Him” (p. 10), “I am not worried... I am in the arms of God “ (p. 14) “... I feel that I am not alone, God is inside me and walks with me” (p. 17).*

**Accepting the divine test**: Some participants believed that illness is a divine test and passing this test is a source of pride: “...sickness and suffering is a divine test for me*... I hope that I will pass this test” (p. 23) ), “...I know that none of God's decisions are without a reason, he tests me” (p. 30).*

## Discussion

The present study explored the spiritual dimension in patients with cancer in four areas of religious needs, strategies to address religious needs, reasons for not using spiritual resources, and impacts of spiritual beliefs. Consistent with the findings of the present study, the results of the other studies showed worshipping and rituals, seeking safety, reflecting and reasoning, as well as communication with and closeness to God (10,24,25), and hopefulness, worshipping, and finding meaning (26) as spiritual needs of patients with cancer. Spirituality, finding meaning, and giving meaning to the aspects of human existence in four routes are based on prayer patterns (27). The themes of religious needs follow this pattern. The desire to worship and perform religious practice is a natural element in human beings and its' mystery should be sought in the inherent human poverty and richness of God (28–30).

This impression helps the patient in the face of unfortunate life events, such as loss or illness, and give him hope for a better life. Following religious practices, a sense of serenity is accomplished, the need for interpersonal relationships is fulfilled, and hope is maintained (10). Reflection and reasoning are the components of spiritual health. Many patients spend quality time comprehending the purpose and transcendent values of their lives. Certainly, if this reflection is done in nature, it will accelerate the recovery process because nature is a sign of His divine superiority and greatness (31). After reflection and spending time with themselves, the patients feel that they are getting their existence from God and He is the only one to be relied on in a physically exhausting condition (6, 32).

Another finding of the study was the strategies to fulfill spiritual-religious needs, including physical activity, achieving inner peace, communication with the medical staff, performing dhikr (Zikr) and prayers, reading religious books and text, making vows, and paying attention and loving others. In other studies, relationships with important people in life, sources of support, prayer and religious rituals, concentration, and exercise have been recognized as important factors in cancer patients' hopefulness (25,33,34). The theme of communication with physicians and nurses in the other studies confirms the outstanding role of healthcare providers in providing emotional support for patients (28,35). The medical staff can provide an affectionate and decent environment to facilitate patients' internal healing power (36).

Patients' religious beliefs can offer support at times of disease, praying, performing rituals, reading religious books, and believing in vows create a positive spiritual feeling for them. By repeating these practices, patients' attitudes change (37). In social psychology, one of the patterns of changing attitudes is praying frequently. Moreover, Dhikr (Zikr) and Salawat can change a person's attitude towards himself, God, creation, and the universe6. Previous studies have also indicated that dhikr (Zikr) and performing rituals are components of spiritual health (38,39). Making vows and reading religious books increases patience in illness and its complications. Additionally, dhikr (Zikr) and praying can be performed in various ways (e.g., reading, listening, speaking) or using techniques, such as meditation, relaxation, and silence. Findings of other studies demonstrated the impact of meditation on increasing pain tolerance and spiritual health, which is consistent with the results of this study (40, 41). In meditation, whether religious or non-religious, one regularly repeats phrases such as “God is peace and serenity,” “God is joy,” and “I am delighted,” which increases resilience in cancer patients. Loving others, praying, and reading religious books are considered efficient resources for patients to increase God's presence in their lives and strengthen their relationship with Him. When a person becomes physically challenged and hospitalized, imagination can enable them to travel to another place where a cure can be found (33).

The reasons for not using spiritual resources were another theme with two sub-themes of lack of belief in the healing powers of praying and believing in a better life after death. To explain this stance, it can be stated that spiritual needs and spirituality are formed by the accepted practices and beliefs of a particular culture. Since each patient possesses unique individual characteristics and different subcultures and backgrounds, it is anticipated that some of them might not need to resort to higher and superhuman forces and then prayers for healing. Due to the differences among individuals, religion practices could build comfort and peace for a person while doing nothing for another. Studies in this field have confirmed the results (30, 42). Additionally, some patients look forward to the otherworld and hope for a better life in the world after death because their religious notions have instilled in them. Culture and individual characteristics influences on religious needs and hope. Nurses and other medical professionals should become more familiar with these factors and the relationship between them. The lack of belief in the healing powers of praying, and on the other hand believing in a better life in the world after death, shows that individual and cultural values determine the path and the meaning and concept of spirituality and religion is not the same for all people (16).

The last theme of this study was the impact of spiritual beliefs. The theme was manifested in four forms, including hope of life, belief in divine destiny, trusting in God's support and accepting the divine test. The findings of the other studies were consistent with the results of the present study (24,40,43). Hopefulness assists patients to cope with a crisis physiologically and emotionally (16, 44). Disappointment is defined as enduring an insurmountable situation in which the achievement of no goal is expected and is associated with depression, the desire to die, and commit suicide (45). Participants reported that belief in divine destiny increases their resilience to the disease and is beneficial in reducing physical, psychological, pain, depression, and anxiety symptoms and boosting hope. Many patients view their illness as a divine test, and believed that if their faith is strong, they will be saved. Evidently, some individuals believe that they are being punished for their past sins and their illness is a test. Consequently, their resilience against the disease increases. Moreover, the patients have come closer to God and believe that they ought to accept whatever God has done to them because He never errs. Therefore, the patients reach a state of peace of mind and do not withstand anxiety and apprehension regarding the future of their illness (36). Life events are unique events and evoke spiritual responses. These events can be an opportunity for cancer patients to use spiritual and religious resources (45) and for nurses to provide spiritual care. Nurses can improve patients' general health by providing nursing support and meeting spiritual needs alongside promoting spiritual health.

The present study was conducted on patients with cancer in a city of Iran with Persian ethnicity and Islam religion. Therefore, further research is suggested in other life threatening disease and in other communities with different religions and cultures.

Exploring the experiences, perceptions, and spiritual needs of cancer patients is of great importance in providing spiritual care. Therefore, nurses should be educated to assess spiritual-religious beliefs and needs of patients in their region to provide and promote holistic care. The findings of the present study, were explored four themes including the spiritual-religious needs, strategies to address spiritual needs, reasons for not using spiritual resources, and the impacts of spiritual beliefs. These findings can help healthcare professionals, especially nurses for effective interaction and communication with patients to address their spiritual needs and accelerate the healing process of patients. However, the findings cannot be generalized in different communities and there is need to conduct more research due to cultural and spiritual/religious differences.
